# 521. Bromelain-based Enzymatic Debridement vs SOC: Post-hoc Analysis of DETECT and CIDS Data on Scar Outcomes

**DOI:** 10.1093/jbcr/irag033.218

**Published:** 2026-04-06

**Authors:** Sean Hickey, Asi Haviv, Daniela Requena, Matthew D Supple, Yaron Shoham, Jeremy Goverman

**Affiliations:** University of California San Diego, San Diego, California; MediWound, Yavne, Israel; Massachusetts General Hospital, Boston, MA; Massachusetts General Hospital, North Reading, MA; Soroka University Medical Center, Beer Sheba, HaDarom; Massachusetts General Hospital, Brookline, MA

## Abstract

**Introduction:**

Long-term scar quality is a key outcome after acute burn care and bromelain-based enzymatic debridement (BBED) utilizing anacaulase-bcdb may reduce the need for surgical autografting and improve scar outcomes in deep partial thickness (DPT) and full thickness (FT) burns.

**Methods:**

A post-hoc pooled, target-wound–level analysis of two, phase III randomized controlled trials (DETECT, adults; CIDS, pediatrics) was performed to evaluate scar outcomes after BBED versus standard of care (SOC). Primary outcomes measures included Modified Vancouver Scar Scale (MVSS) and Patient and Observer Scar Assessment Scale (POSAS) domain and total scores at 12 and 24 months.

**Results:**

Evaluable assessments at 12 and 24 months included MVSS (BBED n = 158, SOC n = 139; BBED n = 113 and SOC n = 99) and POSAS total (BBED n = 160, SOC n = 139; BBED n = 111 and SOC n = 97). At 12 months, pooled MVSS total was lower with BBED (3.88 vs 4.61; *p*=.0304) and POSAS total was lower with BBED (33.92 vs 39.93; *p*=.0087) versus SOC. Differences were driven by non-autografted DPT wounds. At 24 months, MVSS height remained lower with BBED (0.51 vs 0.79; *p*=.0070), with POSAS observer thickness (1.93 vs 2.51; *p*=.0056) and patient overall opinion favoring BBED (2.80 vs 3.54; *p*=.0192).

**Conclusions:**

In pooled phase III data, enzymatic debridement utilizing anacaulase-bcdb achieved superior 12-month scar outcomes versus SOC, particularly in non-grafted DPT burns; and showed domain-level durability at 24 months. These findings, together with consistent adult/pediatric results, support selective eschar removal as a strategy to optimize long-term scar quality.

**Applicability of Research to Practice:**

This study may help inform providers when BBED can be utilized to optimize scar outcomes post-burn injury.

**Funding for the study:**

External Funding - MediWound Ltd, Yavne, Israel.

